# DIFFERENTIAL RESPONSES TO AGING AMONG THE TRANSCRIPTOME AND PROTEOME OF MESENCHYMAL PROGENITOR POPULATIONS

**DOI:** 10.1093/geroni/igaf122.2056

**Published:** 2025-12-31

**Authors:** Jack Feehan, Nicholas Tripodi, Dmitry Kondrikov, Tissa Wijeratne, Jeffrey Gimble, William Hill, Vasso Apostolopoulos, Gustavo Duque

**Affiliations:** Royal Melbourne Institute of Technology, Melbourne, Australia; Victoria University, Melbourne, Victoria, Australia; Medical University of South Carolina, Charleston, South Carolina, United States; Western Health, University of Melbourne, Melbourne, Victoria, Australia; Tulane University School of Medicine, New Orleans, Louisiana, United States; Medical University of South Carolina, Charleston, South Carolina, United States; RMIT University, Melbourne, Victoria, Australia

## Abstract

Cellular senescence, a hallmark of aging, results in a senescence-associated secretory phenotype (SASP) with an increased production of proinflammatory cytokines, growth factors, and proteases. Evidence from nonhuman models demonstrates that SASP contributes to tissue dysfunction and pathological effects of aging. However, there are relatively few human studies on the relationship between SASP and aging-related health outcomes. Proteins from the SASP Atlas were measured in plasma using aptamer-based proteomics (SomaLogic). Regression models were used to identify SASP protein associations with aging-related traits representing multiple aspects of physiology in 1 201 participants from 2 human cohort studies (BLSA/GESTALT and InCHIANTI). Traits examined were fasting glucose, C-reactive protein, interleukin-6, alkaline phosphatase, blood urea nitrogen, albumin, red blood cell distribution width, waist circumference, systolic and diastolic blood pressure, gait speed, and grip strength. Study results were combined with a fixed-effect inverse-variance weighted meta-analysis. In the meta-analysis, 28 of 77 SASP proteins were significantly associated with age. Of the 28 age-associated SASP proteins, 18 were significantly associated with 1 or more clinical traits, and 7 SASP proteins were significantly associated with 3 or more traits. Growth/differentiation factor 15, Insulin-like growth factor-binding protein 2, and Cystatin-C showed significant associations with inflammatory markers and measures of physical function (grip strength or gait speed). These results support the relevance of SASP proteins to human aging, identify specific traits that are potentially affected by SASP, and prioritize specific SASP proteins for their utility as biomarkers of human aging. Several of the identified proteins, specifically of the IGFBP protein family, were also identified as biomarkers of aging in human tissues from healthy aging individuals (as Senescence Network tissue mapping efforts): IBFBP proteins were aging biomarkers in aged human tissues originating from human ovary, spinal cord, and lung.

